# Improving the storage quality and suppressing off-flavor generation of winter jujube by precise micro-perforated MAP

**DOI:** 10.3389/fpls.2024.1372638

**Published:** 2024-04-16

**Authors:** Xinzhi Cui, Yibing Ding, Chanchan Sun, Xiulian Li, Shuzhi Yuan, Fengjun Guo, Xiangquan Zeng, Xinguang Fan, Shuyang Sun

**Affiliations:** ^1^ School of Food Engineering, Yantai Key Laboratory of Nanoscience and Technology for Prepared Food, Yantai Engineering Research Center of Food Green Processing and Quality Control, Ludong University, Yantai, Shandong, China; ^2^ College of Life Sciences, Yantai University, Yantai, Shandong, China; ^3^ School of Pharmacy, Binzhou Medical University, Yantai, Shandong, China; ^4^ Institute of Agri-food Processing and Nutrition, Beijing Academy of Agriculture and Forestry Sciences, Beijing, China; ^5^ Shandong Key Laboratory of Storage and Transportation Technology of Agricultural Products, Jinan, Shandong, China; ^6^ Department of Food Science, College of Agriculture, Purdue University, West Lafayette, IN, United States

**Keywords:** winter jujube, storage quality, volatile component, micro-perforated film, modified atmosphere packaging

## Abstract

**Introduction:**

Traditional modified atmosphere packaging (MAP) cannot meet the preservation requirements of winter jujube, and the high respiration rate characteristics of winter jujube will produce an atmosphere component with high CO_2_ concentration in traditional MAP. Micro-perforated MAP is suitable for the preservation of winter jujube due to its high permeability, which can effectively remove excess CO_2_ and supply O_2_. In this study, a microporous film preservation system that can be quickly applied to winter jujube was developed, namely PMP-MAP (precise micro-perforated modified atmosphere packaging). An experiment was designed to store winter jujube in PMP-MAP at 20°C and 2°C, respectively. The quality, aroma and antioxidant capacity, etc. of winter jujube at the storage time were determined.

**Methods:**

In this study, the optimal micropore area required for microporous film packaging at different temperatures is first determined. To ensure the best perforation effect, the effects of various factors on perforation efficiency were studied. The gas composition within the package was predicted using the gas prediction equation to ensure that the gas composition of the perforated package achieved the desired target. Finally, storage experiments were designed to determine the quality index of winter jujube, including firmness, total soluble solids, titratable acid, reddening, and decay incidence. In addition, sensory evaluation, aroma and antioxidant capacity were also determined. Finally, the preservation effect of PMP-MAP for winter jujube was evaluated by combining the above indicators.

**Results and discussion:**

At the end of storage, PMP-MAP reduced the respiration rate of winter jujube, which contributed to the preservation of high total soluble solids and titratable acid levels, and delayed the reddening and decay rate of winter jujube. In addition, PMP-MAP maintained the antioxidant capacity and flavor of winter jujube while inhibiting the occurrence of alcoholic fermentation and off-flavors. This can be attributed to the effective gas exchange facilitated by PMP-MAP, thereby preventing anaerobic stress and quality degradation. Therefore, the PMP-MAP approach is an efficient method for the storage of winter jujube.

## 1 Introduction

Winter jujube fruit boasts a wealth of vital nutrients (Gao et al., 2013; Bao et al., 2021). Anaerobic respiration can have a negative impact on the quality of winter jujube (Plasquy et al., 2021), including decay and deterioration, resulting in great waste of products (Cheng et al., 2022). Controlled atmosphere (CA) and modified atmosphere packaging (MAP) technology have become the popular methods in the fruit preservation field. These techniques help regulate the atmosphere surrounding the fruit, which can improve storage quality of fruit. Exposing fresh jujube fruit to either 60.0% or 100.0% O_2_ concentration resulted in effective inhibition of MDA accumulation and microbial counts. When stored in MAP at 5°C and 90.0% relative humidity, ‘Phoenix’ variety jujube fruit exhibited consistent appearance over a period of 49 d (Reche et al., 2019).

Micro-perforated MAP has been utilized to preserve the storage quality of fruits, such as durian, strawberry and pear (Xanthopoulos et al., 2012; D Aquino et al., 2017; Boonthanakorn et al., 2020). Micro-perforated film offers the advantages of low processing cost and adaptability to meet the specific preservation requirements of various fruits. It serves as an effective solution for achieving the ideal gas balance in MAP (Hussein et al., 2015; Cai et al., 2017). Some studies have used trial and error to test various films with different perforations, this approach can be inefficient due to the vast number of possible combinations of packaging films and perforation sizes (Kartal et al., 2012; Villalobos et al., 2018; Yuan et al., 2021). Additionally, respiration rates can vary significantly between product batches due to growth conditions and maturity stage (Fonseca et al., 2002). Even if a solution is found to be effective for one batch through trial and error, it may not necessarily work for another (Oliveira et al., 2022). After carefully considering related gas permeance models, we ultimately chose the Del-Valle expression for its simplicity and accuracy (Del-Valle et al., 2009). We have developed a micro-perforated film preservation system that can be quickly applied to different fruit and vegetable, namely PMP-MAP (precise micro-perforated modified atmosphere packaging). Compared to traditional MAP, it can more quickly and accurately form a packaging that will provide an optimum performance.

In this study, we used the Del-Valle expression to calculate the number and size of micropores, then used our self-developed laser perforator machine to create the special microporous film for winter jujube. The application of microporous film as PMP-MAP for winter jujube was further studied at 2°C and 20°C.

## 2 Materials and methods

### 2.1 Preparation of materials

Winter jujubes *Ziziphus jujuba* were obtained from a local farmers’ market in Yantai, China. The fruits were dipped in a water solution containing 100.0 mg L^−1^ chlorine and the fruit surface was gently wiped with a soft sponge. Subsequently, the fruit was rinsed with fresh tap water and left to dry naturally.

### 2.2 Experimental procedure

In this study, the optimal microporous area required for microporous film packaging at different temperatures is first determined. To ensure the best perforation effect, we studied the effects of various factors on perforation efficacy. The gas composition within the packaging was forecasted to change based on the gas prediction equation, ensuring that the desired target was reached by the gas composition of the perforated packaging. Finally, storage experiments were designed to determine the quality index of winter jujube and evaluate the preservation effect of micro-perforated film packaging.

### 2.3 Preparation of micro-perforated film packaging

#### 2.3.1 Preparation of micro-perforated film

We utilized laser drilling to perforate the film. The machine directs a laser beam through an ultra-high-speed scanning vibrating mirror system to achieve marking. It comprises a lifting axis, equipment display, marking vibrating mirror, field mirror, workbench, and computer host. The size of the perforation can be controlled by adjusting the laser focal distance, processing power, and processing times.

#### 2.3.2 Packaging and storage of winter jujube

For the micro-perforated film packaging experiments, a food-grade polypropylene (PP) tray measuring 26.0 cm × 17.0 cm × 8.0 cm was used to pack 700 ± 3 g of winter jujubes fruit. Square food-grade PP plastic containers with a wall thickness of 80.0 μm with permeability of 97.5 mL O_2_ m^−2^ day^−1^ and 84.5 mL CO_2_ m^−2^ day^−1^. To simulate typical supermarket and cold chain transportation conditions, the samples were stored at 20°C and 2°C, respectively, with 85.0-90.0% relative humidity. Based on a literature review, it was determined that the optimal steady-state micro-perforated film packaging conditions for winter jujube fruit are 5.0% O_2_ and 1.0-2.0% CO_2_ (Luo et al., 2021). Three packagings were established for each storage temperature: PP trays heat-sealed on top with either non-perforated modified atmosphere packaging (NP-MAP), precise micro-perforated modified atmosphere packaging (PMP-MAP), or macro-perforated modified atmosphere packaging with 6.0 mm diameter (MP-MAP).

The storage time for the experimental group stored at 20°C was 5 d, and the jujube properties were periodically evaluated every 24 h. The storage time for the experimental group stored at 2°C was 35 d, and winter jujubes were assessed every week on 0 d, 7 d, 14 d, 21 d, 28 d, and 35 d. Each treatment was replicated three times.

### 2.4 Quality assessment of winter jujube

#### 2.4.1 The gaseous compositions inside the package

A portable gas analyzer was utilized to measure changes in the O_2_ and CO_2_ gaseous composition within the headspace of the package (Dansensor, USA).

#### 2.4.2 Respiration rate

The respiratory rate is an important parameter in the formula for calculating the number of micropores on the film. The respiratory rate was measured by method described by Fan et al. (2018). A Telaire 7001 CO_2_/Temperature monitor was used to determine the respiration rate. The winter jujube was stored at a constant temperature of 2°C in a cold room and at 20°C in a constant temperature drying oven to determine the effect of temperature. The winter jujube was placed in an airtight container with a CO_2_ concentration meter to measure CO_2_ production over 1 h, thus determine the respiration rate. A CO_2_ calibration curve was employed and the results were expressed as CO_2_ mg kg^-1^ h^-1^. The measurements were carried out in triplicate.

#### 2.4.3 Weight loss

To determine the weight loss of winter jujubes, measurements were taken of the pulp’s weight before and during the storage period. The percentage loss was then calculated using the following equation:


$$\text{Weight Loss}\left(\%\right)=\frac{W_{0}-W_{t}}{W_{0}}\times 100$$

where $W_{0}$ is the initial weight of the pulp and $W_{\text{t}}$ the weight at time.

(t = 1, 2, 3, 4 and 5 or 7, 14, 21, 28, and 35 d)

#### 2.4.4 Fruit firmness

To determine the firmness of the winter jujube fruit, the BROOKFIELD Texture Analyzer was utilized. This involved applying a force of 0.1 N with a 6.0 mm diameter probe at a cross-head speed of 15.0 mm min^-1^. The probe was inserted 1.0 cm deep into the pulp at a rate of 10.0 mm min^-1^, while the force (N) required to insert the probe was recorded.

#### 2.4.5 Content of total soluble solids (TSS), titratable acid (TA)

To measure the TSS of winter jujube, the pulp was crushed in a mortar to extract the juice. From each of the three packagings, 5.0 g of powdered winter jujube pulp was taken for TSS evaluation. The juice’s TSS was determined using a PAL pocket refractometer (ATAGO, Japan). Similar to the measurement of TSS, the juice for TA measurement was diluted at a 50-fold ratio and assessed using a PAL-BX pocket refractometer (ATAGO, Japan). Measurements of both TSS and TA were conducted with three repetitions for analysis.

#### 2.4.6 Reddening and decay incidence

Three types of packaging for winter jujube are examined for spoilage, with three replicates per packaging type, and the spoilage rate is recorded at each sampling point. Winter jujube fruit is considered spoiled if it exhibits mildew and has a soft, rotten area that constitutes 5.0% of the fruit surface. The decay rate was determined as the percentage ratio of the number of spoiled fruits to the total number of fruits. In addition to the aforementioned decay rate, the reddening rate of winter jujubes was also recorded. A reddening area over 50.0% on the fruit surface was considered as reddened. The reddening rate was calculated as the percentage of reddened fruits to the total number.

#### 2.4.7 Sensory evaluation

A specially trained panel of eight judges assessed the winter jujube by compiling a list of descriptors during preliminary meetings. The focus of sensory analysis centered on examining the crunchiness, juiciness, sweetness, flavor, and visual appeal of the fruits (**Supplementary Table S3**). To quantify the different descriptors, an 8-point intensity scale was employed whereby a score of 0 indicated the absence of the attribute while a score of 8 indicated maximum intensity.

#### 2.4.8 Assessment of total phenolic content (TPC), total flavonoid content (TFC), and antioxidant capacity of winter jujube

3.0 g of winter jujube pulp were blended with 60.0 mL of 70.0% ethanol using a mortar, adapting the method described by Liang et al. (2023). The blend was subjected to ultrasonic extraction for 1 h, followed by 15 min of centrifugation at 16,000 g. The resulting clear liquid was gathered, and the extraction was performed again. The two obtained extracts were merged and condensed to a 10.0 mL volume. Subsequently, this enriched phenolic extract was assessed for its total phenolic content (TPC), total flavonoid content (TFC), and antioxidant potential. To assess the TPC, 1.0 mL of the phenolic extract was mixed with 2.0 mL of Folin-Ciocalteu reagent and 1.0 mL of a 6.0% sodium carbonate solution. This blend was heated in a 70°C water bath for 10 min, then rapidly cooled. The solution’s absorbance was gauged at 765 nm, referencing a blank MP-MAP (where 0.1 mL of phenolic extract was replaced with 0.1 mL of distilled water) using a microplate reader. For the TFC evaluation, 1.0 mL of the phenolic extract was merged with 1.0 mL of distilled water and 0.075 mL of a 5.0% NaNO_2_ solution. After resting for 5 min at ambient temperature, 0.15 mL of a 10.0% AlCl_3_·6H_2_O solution was added, 0.5 mL of 1.0 mol L^-1^ NaOH was introduced. The solution’s absorbance was then gauged at 510 nm with a microplate reader.

The antioxidant capacities of winter jujube were assessed using four tests: ABTS (ABTS free radical scavenging assay), DPPH (DPPH free radical scavenging assay), CUPRAC (cupric ion reducing antioxidant capacity), and FRAP (ferric reducing antioxidant power). For ABTS, 0.03 mL phenolic extract was combined with 5.0 mL of 7.4 mmol L^-1^ ABTS, incubated in darkness for 10 min, and the absorbance measured at 734 nm. In the DPPH test, 1.0 mL of 0.3 mmol L^-1^ DPPH and 0.015 mL phenolic extract were mixed, with a water reference, and incubated at 30°C for 1 hour, absorbance was taken at 517 nm. For CUPRAC, 0.1 mL phenolic extract was mixed with 1.0 mL each of 5.0 mmol L^-1^ CuCl_2_, 3.75 mmol L^-1^ neocuproine, and 1.0 mol L^-1^ CH_3_COONH_4_. After standing at 25°C for 30 min, absorbance was measured at 450 nm. In the FRAP test, 0.2 mL phenolic extract was added to 1.0 mL TPTZ (10.0 mmol L^-1^ in HCl), 1.0 mL FeCl_3_ 6H_2_O (20.0 mmol L^-1^), and 10.0 mL acetate buffer (0.3 mol L^-1^, pH = 3.6) (Liu, 2016). The reaction was incubated at 37°C for 5 min before evaluating the absorbance at 593 nm. The antioxidant capacities were presented in Trolox equivalents. A SpectraMax 190 microplate reader was used to measure the optical density. The TPC and TFC were quantified in terms of gallic acid and rutin equivalents (mg kg^−1^ GAE and RE of FW), respectively, using standard curves.

#### 2.4.9 VOCs analysis by HS-SPME–GC–MS

1.0 g of winter jujube pulp was mixed with 1.0 mL saturated sodium chloride and 3.0 μL of the 2-octanol internal standard (0.01 mg mL^−1^, Aladdin, Heysham, CN). This mixture was placed in a 20.0 mL vial, and a 50/30 μm CAR/PDMS/DVB SPME fiber was used to capture volatiles at 65°C for 30 min. The fiber was then inserted into the GC port at 250°C for 5 min. Volatile components were analyzed using GC-MS, following Cai (2020)’s method beit with minor adjustments. A SHIMADZU GC-2030 with an SH-Stabliwax capillary column (60.0 mm, 0.25 mm i.d., 0.25 µm film) used splitless injection. Helium served as the carrier gas at 1.0 mL min^-1^. The oven started at 40°C for 4 min, increased to 160°C at 5°C min^-1^, then to 230°C at 10°C min^-1^, and held for 10 min. Volatile components were identified against the NIST 2014 and Wiley 8.0 databases. Components’ validation used retention indices and recognized compounds’ spectra. The quantification formula for volatiles was: volatile compound (mg g^-1^) = (compound peak area/standard peak area × standard mass)/sample mass.

#### 2.4.10 Statistical analysis

The statistical analysis of the collected data was performed using Microsoft Office Excel (2019), Originpro 2021 and SPSS 27.0 software. To distinguish the means at a significance level of *P*< 0.05, Duncan’s multiple range test was applied.

## 3 Result

### 3.1 PMP-MAP micropore number calculation

100.0 μm aperture was chosen based on careful consideration. The micropores final transverse spacing was determined to be 50.0 mm and the longitudinal spacing was set to 20.0 mm. The CO_2_ diffusion coefficient in air, storage temperature, respiration rate of winter jujube, initial and equilibrium CO_2_ concentration substituted into the Del-Valle equation:


$$\frac{d\left(N_{O_{2}}\right)}{dt}=\frac{n_{pores}\pi d^{2}}{\left(4x+2d\right)}\times \frac{D_{O_{2,air}\times \left(p_{O_{2}^{out}}-p_{O_{2}^{in}}\right)}}{RT}-WRR_{O_{2}}=0$$

The number of micropores for PMP-MAP at storage temperatures of 20°C and 2°C were calculated to be 20 and 9, respectively.

### 3.2 The influence of machine parameters on the effectiveness of perforation

**Figure 1:**
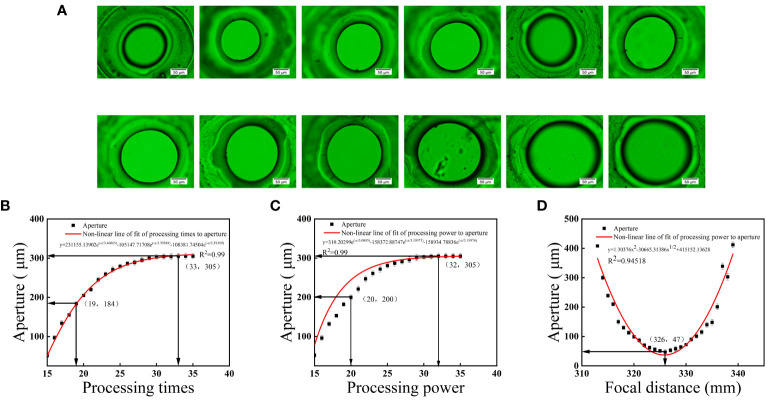
Microscopic images of micropores with different aperture sizes manufactured under different perforation parameters **(A)**, and the effects of processing times **(B)**, processing power **(C)**, and focal distance **(D)** on micropore size, along with the fitting equations.

The pore size in laser perforation is mainly influenced by three factors: the focal distance of the marking vibrating mirror, processing power and times. The interaction of these factors determines the different pore size (**Figure 1A**). To determine the appropriate parameters for achieving the desired pore size, multiple perforation experiments were conducted, ultimately establishing the ideal parameter range, as shown in **Figures 1B–D**. Based on the results of experiments, we have determined that optimal processing power was 102.0 W, processing times was 17, and focal distance was 326.0 mm. Three fitting equations were obtained for processing times, processing power, and focal distance based on these data were $y=-3432e^{-\frac{x}{5.91976}}+293.13875$ , R^2^ = 0.99; y=-3913.15493 $e^{-\frac{x}{5.6181}}+289.90298$ , R^2^ = 0.99; and $y=1.30376x^{2}-30665.31386x^{\frac{1}{2}}+415152.13628$ , R^2^ = 0.94, respectively. These fitting equations have a high degree of fit with the experimental data.

### 3.3 Model validation and evolution of headspace gases

**Figure 2:**
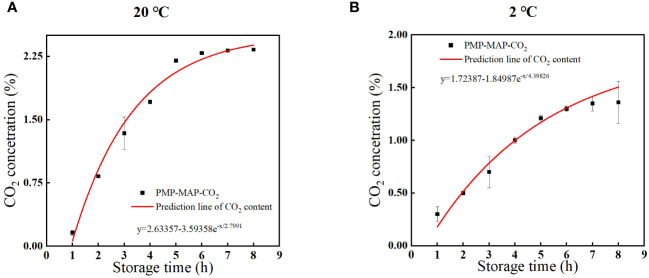
Experimental values of the CO_2_ concentrations in the headspace atmosphere for winter jujube with a PMP-MAP and theoretical evolutions predicted by equation (lines) at 20°C **(A)** and 2°C **(B)**.

As shown in **Figure 2**, the CO_2_ concentration in the PMP-MAP underwent a significant increase during 20°C and 2°C storage and achieved equilibrium 2.2% and 1.5% within 8 h, met the optimal steady-state 1.0-2.0% mentioned above.

The CO_2_ concentration development in the PMP-MAP can be forecasted utilizing a mass balance equation:


$$N_{CO_{2}}\left(t\right)=N_{CO_{2}}\left(0\right)+N_{CO_{2}}\left(permeated\right)+N_{O_{2}}\left(generated\right)$$

After calculation, the prediction models for CO_2_ at 20°C and 2°C are as follows: $y=2.63357-3.59358e^{-\frac{x}{2.7991}}$ with a fitting accuracy of R^2^ greater than 97%; $y=1.72387-1.84987e^{-\frac{x}{4.39826}}$ , with a fitting accuracy of R^2^ greater than 97%. The results showed that the predicted values matched well with the actual values.

**Figure 3:**
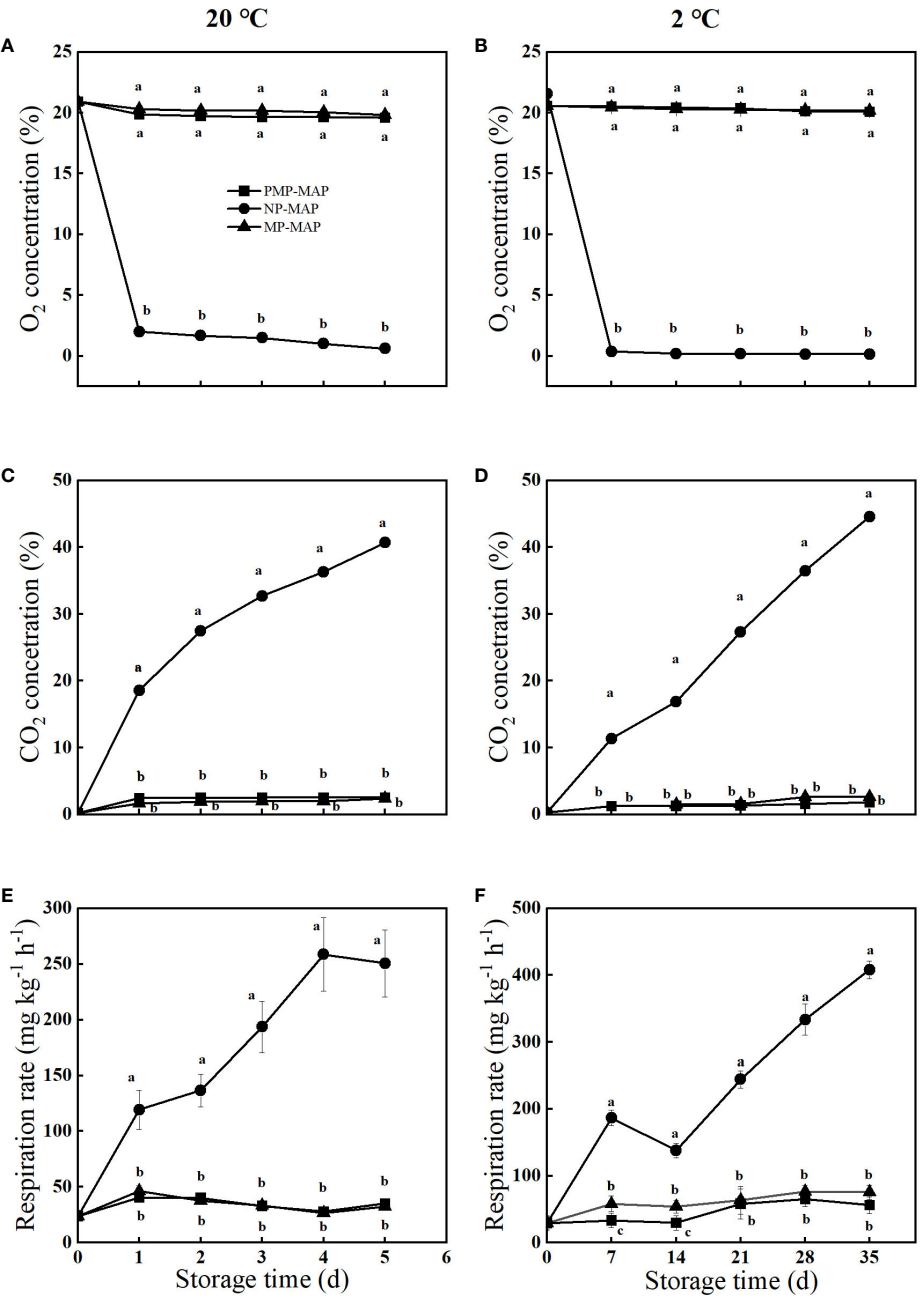
Effects of PMP-MAP on O_2_ concentration **(A, B)**, CO_2_ concentration **(C, D)** and respiration rate **(E, F)** in winter jujube at 20°C and 2°C. Vertical bars represent standard deviations of the means. The different superscripts indicate statistically significant differences between PMP-MAP and MP-MAP, NP-MAP fruit (*P*< 0.05). MP-MAP, macro-perforated MAP; PMP-MAP, precise micro-perforated MAP; NP-MAP, non-perforated MAP.

**Figures 3A–D** shown the evolution of the headspace composition of the three packagings at 20°C and 2°C. In the PMP-MAP, steady state atmosphere of 19.5% O_2_ and 2.2% CO_2_ was achieved and sustained at 20°C. Similarly, a steady state atmosphere of 20.4% O_2_ and 1.5% CO_2_ was reached and maintained at 2°C. Conversely, the CO_2_ concentration in NP-MAP continually increased and was much higher than other two packagings.

### 3.4 Weight loss

**Figure 4:**
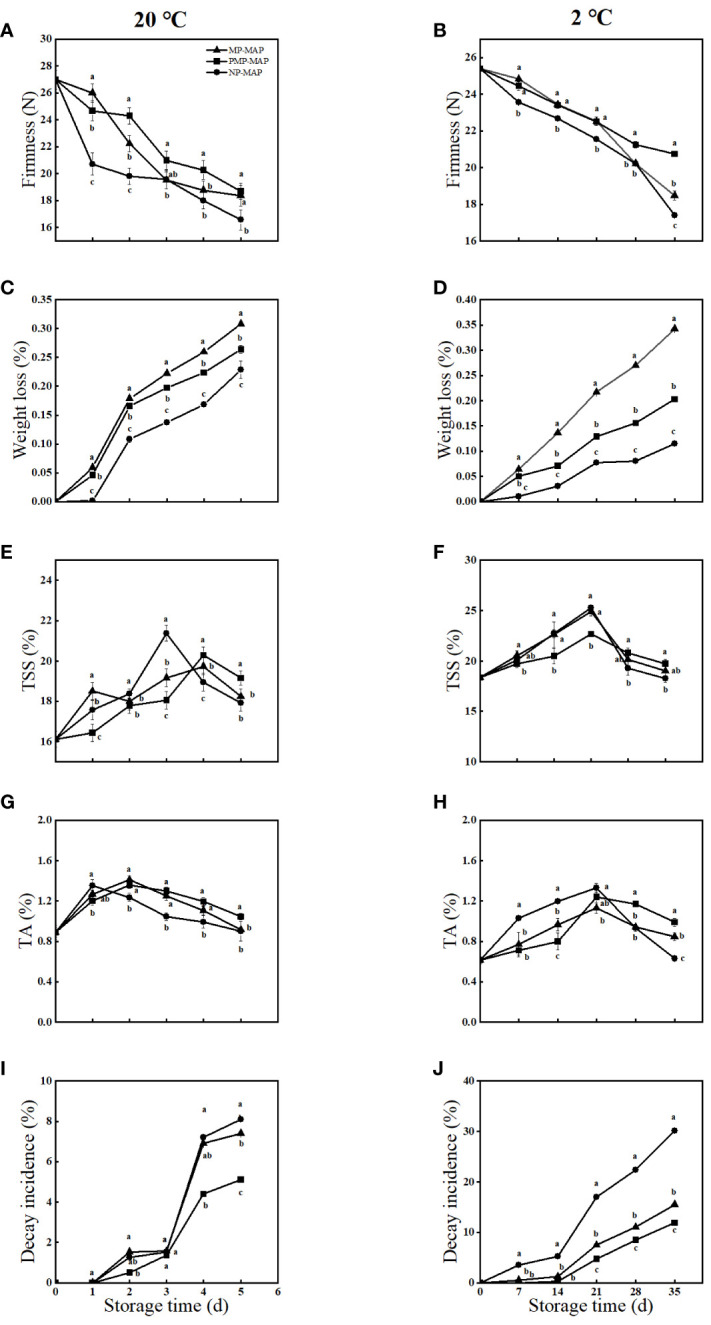
Effects of PMP-MAP, MP-MAP and NP-MAP on firmness **(A, B)**, weight loss **(C, D)**, TSS **(E, F)**, TA **(G, H)** and decay incidence **(I, J)** of winter jujube fruit during shelf life at 20°C and 2 °C. The data presented are the mean values of three replicates; vertical bars represent the standard errors of the mean values; values followed by different superscripts **(A–J)** are significantly different (*P<* 0.05) on the same sampling date. MP-MAP, macro-perforated MAP; PMP-MAP, precise micro-perforated MAP; NP-MAP, non-perforated MAP.

As shown in **Figures 4C, D**. the storage duration extended, the MP-MAP packaged winter jujube exhibited highest weight loss, it was 15.6% higher than PMP-MAP and 50.0% higher than NP-MAP, respectively, at 20°C. During the storage at 2°C, the weight loss of winter jujube in MP-MAP was 50.0% higher than PMP-MAP and 250.0% higher than NP-MAP.

### 3.5 Respiration rate

As shown in **Figures 3E, F**, the respiratory rate of winter jujube in PMP-MAP peaked at 40.8 mg^-1^ kg^-1^ h^-1^ on the 1 d and subsequently stabilized at 35.1 mg^-1^ kg^-1^ h^-1^. The respiration rate of winter jujube in NP-MAP had a rapid increase during storage time, this is 1350.0% higher than PMP-MAP and 1170.0% higher than MP-MAP at 5 d. In another storage temperature (2°C), the change of respiratory rate of winter jujube in three packagings was similar to that of 20°C during storage time. The respiratory rate of winter jujube in NP-MAP was 527.0% higher than PMP-MAP and higher 437.0% than MP-MAP at 35 d.

### 3.6 Firmness

As shown in **Figures 4A, B**, the firmness of winter jujube gradually decreased during the storage period at 20°C. The highest firmness loss of winter jujube was observed in NP-MAP. Fruit firmness in NP-MAP was 16.2% lower than MP-MAP and 17.5% lower than PMP-MAP, respectively, after 5 d of storage. During the storage at 2°C, the firmness of winter jujube fruit continued to decline. The firmness of winter jujube in the PMP-MAP was 15.0% higher than MP-MAP and 26.0% higher than NP-MAP after 35 d. **Supplementary Table S4** showed result of the influence factors cultivar, maturity and their interactions were analyzed using two-way ANOVA.

### 3.7 TSS and TA

As shown in **Figures 4E–H**, the TSS levels of winter jujube in all packagings initially increased and then decreased. The TSS levels of winter jujube in PMP-MAP peaked at 4 d at 20°C, it was 3.0% higher than MP-MAP and 6.8% higher than NP-MAP. The TSS levels of winter jujube in all packagings reached their maximum at 21 d at 2°C. The TSS levels of winter jujube in the NP-MAP surpassed that in the MP-MAP and PMP-MAP by 1.4% and 11.4%. The TA levels of winter jujube initially increased and then decreased during storage time at both temperature conditions. The TA levels of winter jujube in PMP-MAP surpassed that in the MP-MAP and NP-MAP by 16.0% and 16.5% at 5 d at 20°C, respectively, and by 17.0% and 57.1% at 35 d at 2°C.

### 3.8 Reddening and decay incidence

**Figure 5:**
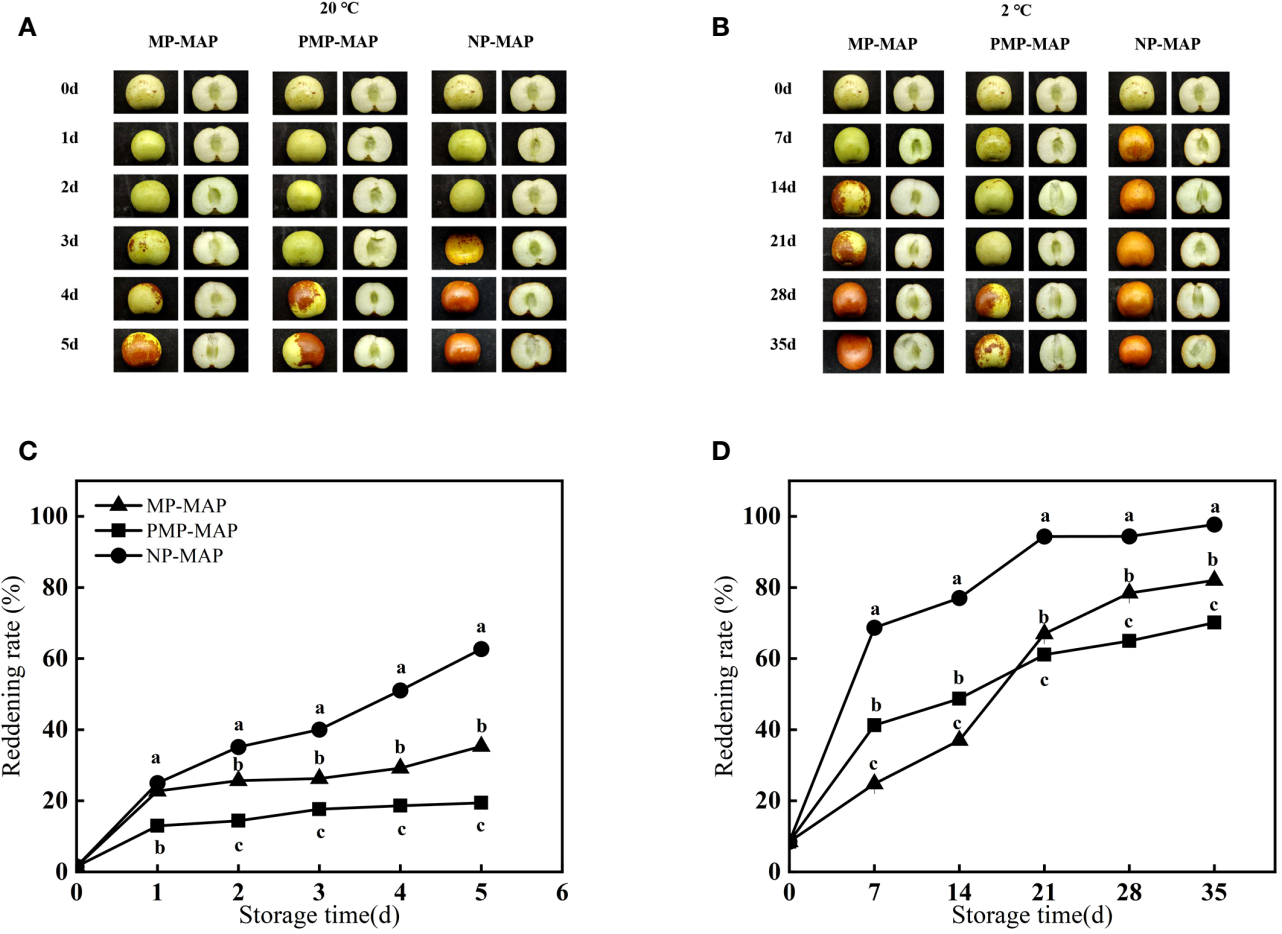
Reddening in winter jujube fruit during different temperature storage **(A, B)** and reddening rate **(C, D)**. MP-MAP, macro-perforated MAP; PMP-MAP, precise micro-perforated MAP; NP-MAP, non-perforated MAP.

As shown in **Figures 5A–D**, the color of the fruit is a significant criterion used by growers and consumers to judge its maturity, ripening stages and grading. The reddening rate of winter jujubes stored in the all packagings exhibited a steady increase and achieved highest values on 5 d at 20°C. The reddening rate of winter jujube in NP-MAP is much higher than the other two packagings. The reddening rate of winter jujube in NP-MAP is up to 97.0% on 35 d at 2°C. As shown in **Figures 4I, J**, with the increase of storage time, the decay incidence of winter jujube also, the greatest decay incidence of winter jujube was observed in NP-MAP at both temperature conditions. The decay incidence in NP-MAP and MP-MAP was 59.0% and 45.1% higher than PMP-MAP at 20°C, respectively, at the end of storage. The decay incidence in NP-MAP and MP-MAP was 150.0% and 29.0% higher than PMP-MAP at 2°C, respectively, at 35 d.

### 3.9 Sensory evaluation

As shown in **Supplementary Table S2**, the winter jujubes stored in PMP-MAP exhibited better quality in terms of crunchiness, juiciness, sweetness, flavor, and visual appearance compared to those stored in the MP-MAP and NP-MAP after 5 d at 20°C. The off-flavor in NP-MAP became more intense with increasing storage time and scored lower in all indicators at 2°C. The fruit’s appearance showed obvious decay, with a significant reduction in the visual appearance score at 35 d. In contrast, both MP-MAP and PMP-MAP scored higher, while PMP-MAP scored higher in visual appearance.

### 3.10 Effect of PMP-MAP on TPC, TFC and antioxidant capacities of winter jujube

**Figure 6:**
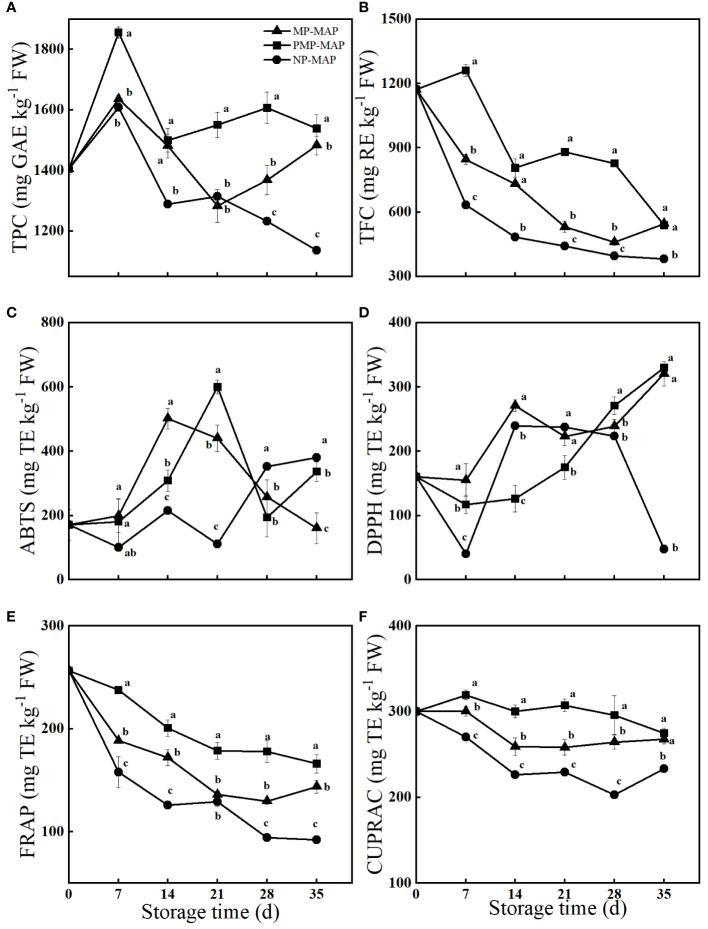
Effect of PMP-MAP on total phenolic content (TPC, **A**), total flavonoid content (TFC, **B**), ABTS free radical scavenging assay (ABTS, **C**), DPPH free radical scavenging assay (DPPH, **D**), Ferric reducing antioxidant power assay (FRAP, **E**) and Cupric ion reducing antioxidant capacities assay (CUPRAC, **F**) of winter jujube during cold storage at 2°C for 35 d. TPC is reported in gallic acid equivalent (GAE), TFC is reported in rutin equivalent (RE), and antioxidant capacities are reported in trolox equivalents (TE). Bars represent standard deviation of the mean. Different letters above the bars within the same time indicate significant difference at *P*< 0.05 level. MP-MAP, macro-perforated MAP; PMP-MAP, precise micro-perforated MAP; NP-MAP, non-perforated MAP.

As shown in **Figure 6**, the TFC of winter jujube consistently declines with extended storage duration. Winter jujubes stored in PMP-MAP exhibited high TFC, surpassing the levels of the other two packagings. A similar trend was observed in TPC, which winter jujube in PMP-MAP consistently exhibited the highest TPC throughout the storage duration. The DPPH scavenging activity of winter jujube in PMP-MAP increased during storage, which was 598.4% higher than that of NP-MAP packaged winter jujube at 35 d. The ABTS scavenging activity of winter jujube in PMP-MAP peaked at 21 d, it was 36.2% higher than MP-MAP and 442.9% higher than NP-MAP. The CUPRAC of PMP-MAP packaged winter jujube was almost higher than other packagings all the storage time. The CUPRAC of winter jujube in PMP-MAP was 20.9% higher than MP-MAP and 33.9% higher than NP-MAP at 21 d. In addition, the FRAP of winter jujube packaged in PMP-MAP was respectively 15.4% and 80.4% higher than that of winter jujube packaged in MP-MAP and NP-MAP at 35 d.

### 3.11 PCA of jujube fruits at different packagings

**Figure 7:**
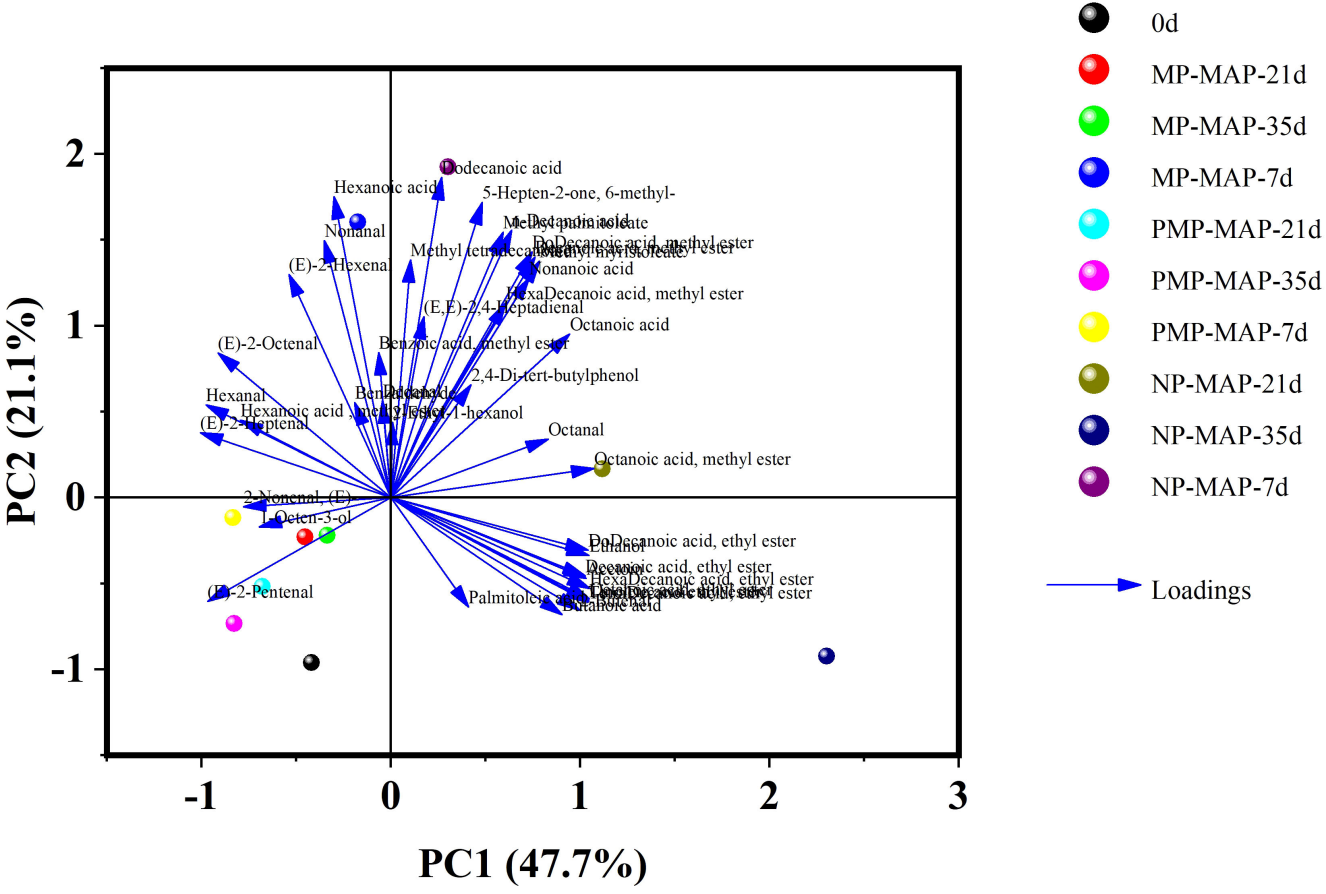
PCA analysis of winter jujube. Biplot of the first two principal components, MP-MAP= macro-perforated MAP; PMP-MAP= precise micro-perforated MAP; NP-MAP= non-perforated MAP. 0 d, 7 d, 21 d, 35 d represent storage at 2°C for 0, 7, 21, and 35 d, respectively.

As shown in **Figure 7**, the first and second principal components (PC1 and PC2) contributed to 47.7% and 21.1% of the total variance respectively. The winter jujubes in PMP-MAP were distinctly segregated from those in other packaging types, demonstrating significant variations in aroma composition, particularly in (E)-2-Nonenal, 1-octen-3-ol, and (E)-2-Pentenal—common aromas in winter jujube. Compared to NP-MAP, the aroma of PMP-MAP packaged jujube at each storage interval is closer to the baseline, signifying that PMP-MAP has a better efficacy in preserving winter jujube’s aroma and flavor. With extended storage time, winter jujubes in NP-MAP produced a significant amount of absent VOCs (Volatile Organic Compounds) in fresh winter jujube, primarily esters, aldehydes, and acids included off-flavor substances such as decanoic acid ethyl ester, ethanol, butanoic acid, and pentanoic acid, etc. odorant.

### 3.12 Cluster analysis of VOCs of winter jujube fruits from different periods and packaging based on the heat map

**Figure 8:**
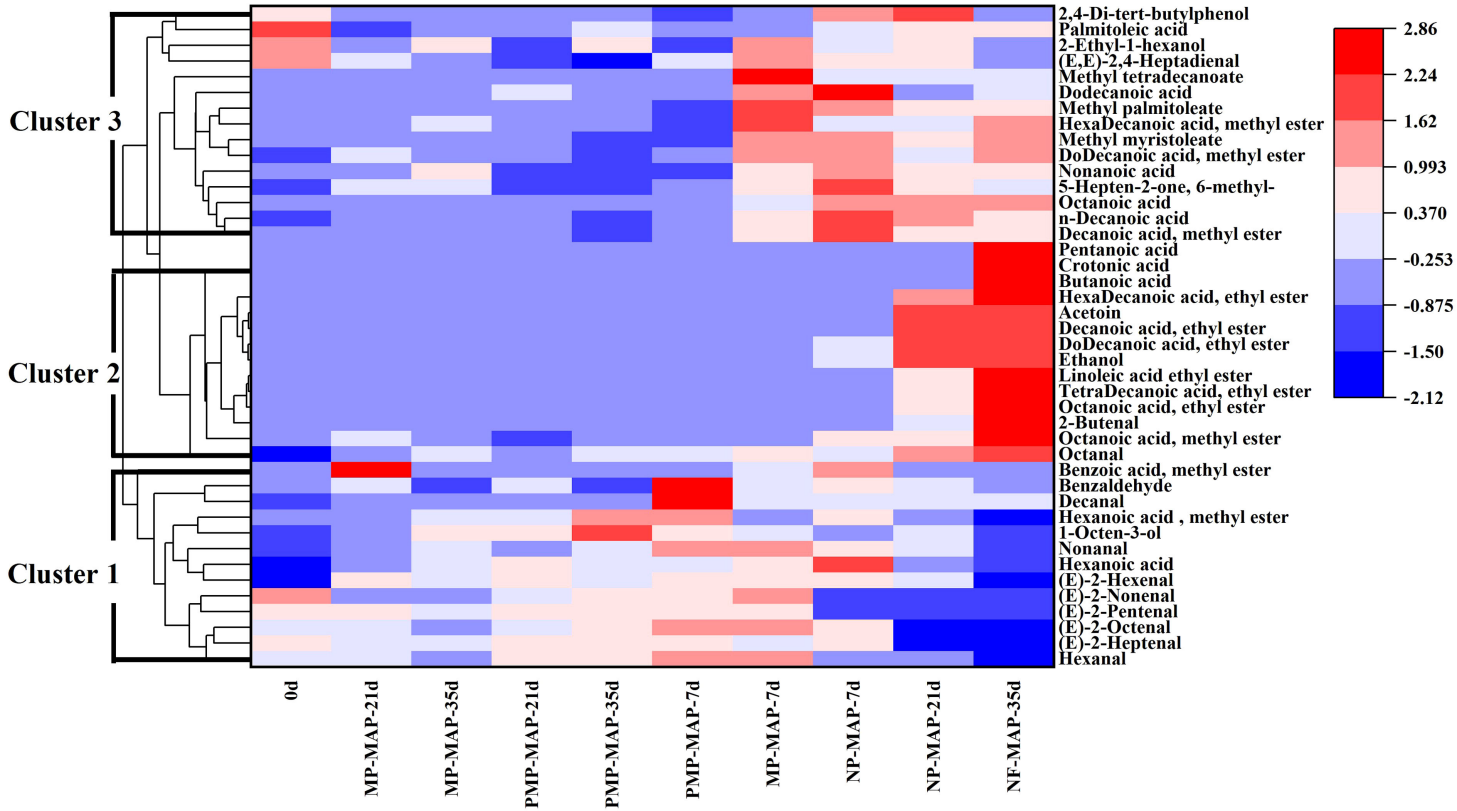
Heat map and cluster analysis of winter jujube at different periods. MP-MAP, macro-perforated MAP; PMP-MAP, precise micro-perforated MAP; NP-MAP, non-perforated MAP. 0 d, 7 d, 21 d, 35 d represent storage at 2°C for 0, 7, 21, and 35 d, respectively.

To assess the differences in VOCs among winter jujube fruits stored in diverse packaging over varying durations, a cluster analysis was performed, visualized using a heatmap, and the total ion chromatogram is displayed in **Supplementary Table S1**. In **Figure 8**, the color gradient from blue to red signifies the change in VOCs content from low to high. According to the vertical direction of the heat map, all samples were classified into three main categories. The cluster 1 comprises the VOCs responsible for the distinct aroma of winter jujube, including hexanal, (E)-2-pentenal, (E)-2-hexenal, (E)-2-heptenal, and 1-octen-3-ol, etc. In PMP-MAP, a color composition dominated by light red and crimson shades in cluster 1 was observed, indicates a high content of VOCs in cluster 1 in winter jujube stored in PMP-MAP. The MP-MAP section exhibited less red. Instead, it is primarily dominated by light blue, indicating these VOCs are less prevalent in MP-MAP packaged winter jujube compared to PMP-MAP. Notably, NP-MAP displayed a deeper and more extensive blue, indicating deficiency in the content of VOCs in cluster 1 in winter jujube packaged with NP-MAP. The VOCs in cluster 2, rich in acids, esters and alcohol, such as pentanoic acid, crotonic acid, butanoic acid, hexadecanoic acid ethyl ester, decanoic acid ethyl ester, dodecanoic acid ethyl ester and ethanol, etc. These are likely to comprise the primary VOCs responsible for the off-flavor. In **Figure 8**, both PMP-MAP and MP-MAP predominantly displayed blue, suggesting that the content of VOCs in cluster 2 is less in these packagings. In contrast, NP-MAP begins with a light blue at 7 d of storage, deepening to light red and even crimson as storage time extends. This progression indicated a rising content of VOCs in cluster 2 of winter jujubes packaged in NP-MAP. The cluster 3 of VOCs, similar to cluster 2, is also rarely found in fresh winter jujube fruits. Mainly composed of acids and esters, including octanoic acid, nonanoic acid, palmitoleic acid, methyl tetradecanoate, dodecanoic acid methyl ester, hexadecanoic acid methyl ester, etc. PMP-MAP exhibits color of dark blue, blue, and light red, whereas MP-MAP presents a mix of blue, light red, and sporadic crimsons. This color distribution implied lower content of VOCs in cluster 3 of winter jujubes within these two packages. Meanwhile, NP-MAP consistently shows red and crimson, denoting higher content of VOCs in cluster 3.

## 4 Discussion

In this study, an PMP-MAP for jujube preservation was designed and manufactured using laser perforation machine and a scientific and effective perforation equation. Unlike previous single-factor MAP experiments, this study no longer focused on the effect of different micropore numbers or diameter on the freshness of fruits. Instead, it attempts to establish a scientific and efficient PMP-MAP development and application system, from packaging design to packaging application. The CO_2_ concentration observed in winter jujubes were found to be very close to the recommended levels (1.0-2.0%) when stored in PMP-MAP. This is due to the capability of micropores in regulating the concentrations of O_2_ and CO_2_ inside the packaging. In contrast, NP-MAP without micropores can only exchange gas through film permeation, leading to the accumulation of a large amount of CO_2_. Such observations clearly suggest the advantage of PMP-MAP for winter jujube storage over NP-MAP (Zhang et al., 2015). As shown in **Figure 3**, the reduction in O_2_ is almost equal to the increase in CO_2_ in both PMP-MAP and the MP-MAP, indicating that the winter jujube in these two packages maybe undergoing aerobic respiration. However, the increase in CO_2_ concentration in the NP-MAP at 20°C and 2°C was 20.0% and 27.0% higher, respectively, than the decrease in O_2_ concentration, it indicates that winter jujube in NP-MAP may undergo a transition from aerobic respiration to anaerobic respiration after the O_2_ in the packaging is depleted. This ultimately led to quality degradation of winter jujube. The firmness of winter jujube preserved in PMP-MAP surpassed that in MP-MAP. Muftuoglu et al., (2012) found that firmness variations in apricots stored under MAP with PP film correlated with moisture evaporation from the packaging over an 18 d storage at 5°C. The most pronounced weight loss in the MP-MAP can be explained by the prominent pores in the MP-MAP, which might have accelerated the dehydration of winter jujube. TSS levels in winter jujube fruit initially increase and then decrease. This phenomenon could be attributed to the existence of large molecular substances, such as starch, in the fruit during the initial stages of its shelf life (Zhang W. et al., 2022). The decomposition of these compounds might boost the early-stage TSS levels in winter jujube fruit during its initial shelf life. Yet, as shelf-life progresses, TSS levels might diminish owing to nutrient utilization from respiration. The slowest increase and decrease in TSS levels of winter jujube in PMP-MAP may be due to the appropriate gas composition in their packaging inhibiting the respiratory rate of the winter jujube. This could slow down the degradation of large molecular substances and inhibit nutrient consumption due to respiration at the later stage of shelf life, thus maintaining high TSS levels of winter jujube. The high respiratory rate in NP-MAP may have contributed to the low TSS levels of winter jujube. The organic acids of winter jujube fruits act as vital substrates for respiration, bolstering its metabolism and profoundly influencing its taste characteristics. During the middle and later stages of shelf life, it was increasingly utilized as a respiratory substrate, TA levels declined out of all the various types of packaging, the use of PMP-MAP resulted in higher levels of TA in winter jujube than the MP-MAP and NP-MAP. This is a key factor, as maintaining TA levels during storage is crucial in preserving the taste of freshly-harvested winter jujube fruit (Baswal et al., 2020). This is also consistent with the high flavor score of winter jujube in PMP-MAP in the sensory evaluation. During the process of fruit ripening, the ethylene and CO_2_ can quickly diffuse out through the micropores, avoiding further damage to the winter jujube. This helped increase TSS and prevent a rapid decline in TA levels, consequently delaying fruit senescence. The reddening of the pericarp of winter jujube is a key indicator of the fruit’s ripeness. The rate of reddening in winter jujube packaged in PMP-MAP was lower than that in NP-MAP and the MP-MAP, especially under refrigeration conditions. The winter jujube in NP-MAP showed a reddening that was different from the normal ripening of winter jujube and more similar to browning. This abnormal color change might due to physiological damage caused by anaerobic stress (Zhang M. et al., 2022). As the CO_2_ concentration in the packaging increases, it enhances the anaerobic respiration of winter jujubes and triggers ethanol fermentation, leading to the spoilage of the fruit pulp, softened and a strong odor of alcohol, which negatively impacts the fruit’s sensory quality (Li, 2003). During storage, the decline in the sensory quality of winter jujube occurred in all packagings. After 5 d, winter jujube stored in PMP-MAP had better quality in terms of crunchiness, juiciness, sweetness, flavor, and visual appearance compared to NP-MAP at 20°C, overall score is also high (**Supplementary Table S2**). The differences were more pronounced at cold storage temperatures. There was little difference at 20°C compared to the MP-MAP, but a greater difference under refrigeration, which may be due to the longer storage time of cold storage. At the end of the storage period, it was observed that the winter jujube stored under the MP-MAP and NP-MAP showed lower fruit quality compared to the PMP-MAP. The sensory evaluation of overall quality, acidity, and sweetness showed that winter jujube stored in PMP-MAP had better quality attributes than those that were stored using traditional packaging methods.

As storage time extends, jujubes stored at 2°C begin to develop a unique wine-like aroma and display symptoms akin to internal browning. Consequently, the antioxidant capacities and VOCs of these winter jujubes continue to be closely analyzed and defined. The antioxidant potential of fruit could by its ability to scavenge radicals and its reduction capability (Fan et al., 2021; Liang et al., 2023). As shown in **Supplementary Table S5**. The TPC and TFC of winter jujube were strongly correlated with CUPAC and FRAP (CUPAC: R = 0.816 for TPC; R = 0.728 for TFC; FRAP: R = 0.849 for TPC; R = 0.952 for TFC). The TPC and TFC of winter jujube packaged in PMP-MAP were consistently higher than those in both MP-MAP and NP-MAP during the storage duration. Compared to the other two packagings, winter jujube in PMP-MAP exhibited a higher antioxidant capacity. PMP-MAP had a more pronounced effect on maintaining CUPAC and FRAP of winter jujube than both MP-MAP and NP-MAP, winter jujube packaged in PMP-MAP exhibit enhanced antioxidant capacities. Conversely, winter jujube packaged in NP-MAP displayed the lowest CUPAC and FRAP values and this diminished antioxidant capacity might render them more vulnerable to membrane lipid peroxidation and internal browning. However, the underlying molecular mechanism is still ambiguous and warrants deeper investigation (Liu et al., 2022).

Through PCA and heatmap analysis, the differences in types and content shifts of VOCs in winter jujubes across various packaging types are evident. Compared with MP-MAP and NP-MAP, PMP-MAP maintained the flavor and aroma of winter jujube during the whole storage period, and the content of hexanal, (E)-2-pentenal, (E)-2-hexenal, (E)-2-heptenal, and 1-octen-3-ol, etc. were higher than those of MP-MAP and NP-MAP packaged winter jujube. These compounds are all important components of the aroma in winter jujube (Chen et al., 2018). In contrast, the content of the above aroma substances in winter jujube from NP-MAP and MP-MAP was lower and had a significant decreasing trend. Hexanal having fruity, grassy, and green flavors and a low odor threshold of 1.1 ng L^-1^ (Hu et al., 2020). (E) -2-nonenal, a plant metabolite derived from linoleate decomposition, possesses a paper-like flavor. (E) -2-heptenal, a plant metabolite exuding a soapy and fatty aroma, is found in the peel of white pomelo (De-la-Fuente-Blanco et al., 2020). These compounds are the principal components of cluster 1 of VOCs and play a significant role in the aroma and flavor profile of fresh winter jujube. Notably, most of these common volatiles contribute to the fruit’s distinctive aroma attributes, predominantly fruity, grassy, or green. The VOCs in cluster 2 and 3, rich in acids, esters and alcohol, such as octanoic acid ethyl ester, decanoic acid ethyl ester, tetra decanoic acid ethyl ester, butanoic acid, crotonic acid, and ethanol. These could potentially be fermentative metabolites produced under the high CO_2_ conditions within the NP-MAP, which result in undesirable flavors and odors (Zhao et al., 2016; Kamatou and Viljoen, 2017). It may also be associated with browning due to CO_2_-stress cell membrane damage. This may explain the unnormal reddening of winter jujube in NP-MAP (Li et al., 2022). Notably, the presence of ethanol is distinct marker of winter jujube alcoholism, aligns with the intense boozy aroma emanating from the NP-MAP stored winter jujubes (Tudela et al., 2013). Additionally, the specific acids and esters found in NP-MAP packaged winter jujubes correlate with those documented in literature pertaining to the alcoholic fermentation of winter jujube (Cai et al., 2020). Therefore, winter jujubes in PMP-MAP can be preserve their flavor. Additionally, the suitable gas environment within this packaging also inhibits the potential occurrence of alcoholic fermentation and the emergence of off-flavors in the winter jujubes.

## 5 Conclusion

In this study, the Del-Valle model and Fick’s law were utilized to optimize the processing parameters of PMP-MAP for winter jujube. The micropore diameter was determined to be 100.0 μm and the optimal number of micropores were found to be 20 for 20°C storage and 9 for 2°C storage. The processing parameters were determined as 17 of processing times, 102.0 W of processing power, and 326.0 mm of focal distance. PMP-MAP could reduce the respiratory rate of winter jujube which contributed to the preservation of high TSS and TA levels, and delay reddening and decay rate of winter jujube. Furthermore, PMP-MAP maintained the antioxidant capacities and flavor of winter jujube, while also inhibiting the occurrence of alcoholic fermentation and off-flavors. This can be attributed to the effective gas exchange facilitated by PMP-MAP, which expels excess CO_2_ and replenishes O_2_, thereby preventing anaerobic stress and quality degradation. Therefore, the approach for PMP-MAP is an efficient method for the storage of winter jujube.

## Data availability statement

The original contributions presented in the study are included in the article/**Supplementary material**. Further inquiries can be directed to the corresponding authors.

## Author contributions

XC: Writing – review & editing. YD: Writing – review & editing, Conceptualization. CS: Conceptualization, Writing – review & editing. XL: Conceptualization, Writing – review & editing. SY: Software, Writing – review & editing. FG: Methodology, Writing – review & editing. XZ: Supervision, Writing – review & editing. XF: Funding acquisition, Writing – review & editing. SS: Writing – review & editing.
