# Supplementary material for: Improving the storage quality and suppressing off-flavor generation of winter jujube by precise micro-perforated MAP
Source: Front Plant Sci. 2024 Apr 16;15:1372638. doi: 10.3389/fpls.2024.1372638 (PMC11058657; doi:10.3389/fpls.2024.1372638)
Supplement: Supplementary file 1 [file Table_1.docx]

Table S1 Concentration of volatile compounds in jujube fruit by different packagings at 2 ^o^C.

| RT (s)^2^ | Compounds | RI (s)^2^ | Content of volatile compounds in jujube fruit (μg/kg) | | | | | | | | | |
| --- | --- | --- | --- | --- | --- | --- | --- | --- | --- | --- | --- | --- |
|  |  |  | 0 d | 7 d | | | 21 d | | | 35 d | | |
|  |  |  |  | MP-MAP | PMP-MAP | NP-MAP | MP-MAP | PMP-MAP | NP-MAP | MP-MAP | PMP-MAP | NP-MAP |
|  | **Alcohols** |  |  |  |  |  |  |  |  |  |  |  |
| 1451.1 | 1-Octen-3-ol | 1450.0 | 2.0±0.03a | 3.1±0.5a | 2.2±0.5a | 2.4±0.5a | 1.7±0.2a | 1.8±0.2a | 2.3±0.2a | 2.5±0.5a | 2.7±0.3a | 1.6±0.04b |
| 1489.8 | 2-Ethyl-1-hexanol | 1491.0 | 3.1±0.2a | 2.7±0.2a | 2.3±0.0a | 1.9±0.0a | 2.4±0.0a | 2.1±0.2a | 2.4±0.3a | 3.1±0.1a | 2.3±0.0a | 2.7±0.0a |
| 1198.1 | Ethanol | 932.0 | _ | _ | _ | 25.5±3.5 | _ | _ | 182.9±12.6 | _ | _ | 198.6±6.6 |
|  | **Acid** |  |  |  |  |  |  |  |  |  |  |  |
| 1779.5 | Hexanoic acid | 1846.0 | 41.0±3.8a | 106.9±1.2b | 49.7±4.5a | 122.3±1.1c | 57.2±8.4a | 61.5±2.9a | 49.6±14.3a | 76.3±17.1c | 53.8±5.1b | 33.1±1.5a |
| 1965.5 | Octanoic acid | 2060.0 | 3.4±1.1a | 7.9±1.9b | 3.8±0.4a | 7.7±1.3b | 3.3±0.5a | 3.6±0.1a | 6.2±1.2b | 4.1±0.6b | 3.2±0.5a | 8.7±0.1c |
| 2087.7 | n-Decanoic acid | 2276.0 | 24.7±2.5a | 51.9±5.2b | 23.5±5.3a | 68.9±14.2b | 22.9±5.9a | 31.6±3.7ab | 44.6±14.8b | 19.7±3.3a | 14.5±1.4a | 39.0±4.4b |
| 2193.1 | Dodecanoic acid | 2497.0 | 19.1±1.6a | 38.7±4.1b | 15.3±3.0a | 49.7±13.8b | 17.1±4.4a | 22.5±4.2a | 26.4±10.8a | 14.3±2.5a | 11.6±1.2a | 26.8±2.8b |
| 2034.6 | Nonanoic acid | 2171.0 | 4.1±0.8a | 4.8±1.5b | 1.6±0.4a | 3.9±0.8b | 1.6±0.4a | 1.4±0.3a | 2.8±1.0b | 4.2±1.3b | 1.3±0.2a | 3.0±0.8a |
| 2523.2 | Butanoic acid | 1625.0 | _ | _ | _ | _ | _ | _ | _ | _ | _ | 5.9±0.5 |
| 1815.6 | Pentanoic acid | 1733.0 | _ | _ | _ | _ | _ | _ | _ | _ | _ | 28.7±3.4 |
| 2629.1 | Crotonic acid | 1745.0 | _ | _ | _ | _ | _ | _ | _ | _ | _ | 3.6±0.8 |
| 1870.6 | Palmitoleic acid | 2926.0 | 2.5±0.3a | 0.9±0.1a | 0.8±0.1a | 1.6±0.3b | 0.4±0.1a | 1.0±0.1b | 1.3±0.5b | 1.2±0.2b | 0.7±0.1a | 1.7±0.1c |
|  | **Esters** |  |  |  |  |  |  |  |  |  |  |  |
| 1178.3 | Hexanoic acid,  methyl ester | 1184.0 | 19.7±4.3a | 37.0±1.4a | 28.0±12.0a | 37.3±1.8a | 23.5±1.1a | 29.1±3.1a | 25.4±3.8a | 44.0±2.1b | 34.5±2.0b | 15.5±3.2a |
| 1369.2 | Octanoic acid,  methyl ester | 1385.0 | 1.3±0.1a | 1.7±0.3a | 1.7±0.6a | 2.3±0.1a | 1.4±0.3a | 1.2±0.2a | 1.9±0.3b | 1.5±0.1a | 1.3±0.2a | 3.1±0.3b |
| 1599.3 | Decanoic acid,  methyl ester | 1593.0 | 17.7±4.7a | 24.4±4.0a | 18.4±1.3a | 36.3±6.4b | 16.7±1.1a | 17.6±2.4a | 27.2±4.1b | 16.9±0.1b | 10.8±0.8a | 28.8±3.6c |
| 1641.7 | Benzoic acid,  methyl ester | 1612.0 | 28.4±1.2a | 33.8±1.1b | 20.8±1.4a | 55.9±7.1c | 93.8±1.5c | 23.9±2.9b | 19.2±0.0a | 15.2±1.7a | 15.5±0.6a | 21.6±2.8b |
| 1759.2 | Dodecanoic acid, methyl ester | 1804.0 | 22.0±6.4a | 28.9±1.8a | 20.9±1.2a | 40.5±1.3b | 19.8±3.1 | 20.2±2.5 | 22.4±1.4 | 17.9±2.2 | 14.0±0.6 | 27.7±4.6 |
| 1915.2 | Methyl tetradecanoate | 2005.0 | 3.8±0.1a | 6.9±0.7b | 3.2±1.0a | 7.0±1.4b | 3.3±0.6a | 3.5±0.4a | 4.3±1.2a | 4.1±0.6a | 3.7±0.8a | 6.1±0.6b |
| 1957.4 | Methyl myristoleate | 2026.0 | 6.5±0.8a | 11.3±1.0a | 7.1±0.9a | 13.2±1.7b | 6.6±1.2a | 7.0±0.6a | 10.8±0.1b | 5.9±0.7a | 5.6±0.9a | 11.4±0.6b |
| 2060.5 | Hexadecanoic acid，  methylester | 2208.0 | 1.4±0.5a | 3.0±0.6b | 1.3±0.7a | 2.0±0.4a | 1.1±0.2a | 1.2±0.1a | 2.4±0.1b | 1.8±0.3a | 1.6±0.4a | 2.9±0.3b |
| 2076.1 | Methyl palmitoleate | 2240.0 | 7.0±0.8a | 17.0±1.4b | 7.0±0.7a | 12.3±0.9b | 6.4±0.00a | 6.3±1.0a | 10.7±0.7b | 8.6±1.0a | 7.7±0.7a | 13.2±0.6b |
| 2232.1 | Octanoic acid, ethyl ester | 1435.0 | _ | _ | _ | _ | _ | _ | 3.5±0.4 | _ | _ | 7.6±0.4 |
| 2533.2 | Decanoic acid, ethyl ester | 1638.0 | _ | _ | _ | _ | _ | _ | 39.3±3.6 | _ | _ | 42.4±0.9 |
| 2893.1 | Tetradecanoic acid, ethyl ester | 2049.0 | _ | _ | _ | _ | _ | _ | 7.0±0.00 | _ | _ | 14.0±2.5 |
| 3004.1 | Hexadecanoic acid, ethyl ester | 2251.0 | _ | _ | _ | _ | _ | _ | 2.3±0.3 | _ | _ | 2.5±0.1 |
| 3182.2 | Linoleic acid ethyl ester | 2521.0 | _ | _ | _ | _ | _ | _ | 0.5±0.1 | _ | _ | 1.0±0.2 |
| 2754.2 | Dodecanoic acid, ethyl ester | 1841.0 | _ | _ | _ | 7.0±0.4 | _ | _ | 40.6±1.5 | _ | _ | 62.9±1.3 |
|  | **Ketones** |  |  |  |  |  |  |  |  |  |  |  |
| 1343.6 | 5-Hepten-2-one, 6-methyl- | 1338.0 | 4.0±0.9a | 4.3±0.3a | 3.4+0.3a | 4.3±0.7a | 3.4±0.3b | 2.7±0.5a | 2.3±0.1a | 3.3±0.6a | 3.3±0.4a | 3.5±0.2a |
| 1974.2 | Acetoin | 1284.0 | _ | _ | _ | _ | _ | _ | 19.6±0.4 | _ | _ | 23.8±0.1 |
|  | **Aldehydes** |  |  |  |  |  |  |  |  |  |  |  |
| 1053.4 | Hexanal | 1083.0 | 16.6±0.5a | 27.3±0.7a | 20.9±0.4a | 21.4±0.6a | 23.3±0.7a | 20.1±0.6a | 12.9±0.4b | 18.4±0.3a | 23.1±1.7a | _ |
| 1122.5 | (E)-2-Pentenal | 1127.0 | 13.1±1.8a | 7.7±1.0a | 11.4±0.3b | _ | 12.4±1.5a | 10.1±0.9a | _ | 9.9±1.1a | 11.7±0.3a | _ |
| 1200.6 | (E)-2-Hexenal | 1216.0 | 2.7±0.2a | 3.2±0.3a | 3.3±0.2a | 3.7±0.6a | 3.4±0.5a | 2.6±0.2b | 2.0±0.0b | 2.4±0.5a | 2.3±0.5a | 15.7±1.7b |
| 1290.6 | Octanal | 1289.0 | 1.5±0.1a | 2.9±0.6a | 6.3±0.1b | 3.0±0.9a | 2.6±0.7a | 2.3±0.3a | 3.1±0.5a | 2.1±1.0a | 2.7±0.3ab | 3.2±0.1b |
| 1332.9 | (E)-2-Heptenal | 1323.0 | 6.8±0.7a | 5.7±1.2a | 6.8±0.4a | 6.2±0.1a | 6.4±0.9a | 5.6±1.1a | 2.2±0.3b | 5.3±0.3a | 6.7±0.5a | 1.6±0.1b |
| 1399.0 | Nonanal | 1391.0 | 5.5±0.4a | 11.7±0.5a | 15.0±0.5a | 8.3±2.7a | 6.5±0.5a | 5.4±0.2a | 8.8±0.3a | 8.6±0.7a | 8.1±0.1a | 5.8±0.9b |
| 1441.6 | (E)-2-Octenal | 1429.0 | 4.5±0.2a | 5.0±0.7a | 5.4±0.3a | 4.1±0.2a | 4.0±0.6a | 3.8±0.0a | 2.0±0.4b | 4.0±0.1a | 4.7±0.4a | 2.0±0.1b |
| 1478.2 | (E,E)-2,4-Heptadienal | 1495.0 | 2.6±0.5a | 1.2±0.2a | 2.0±0.1b | _ | 2.2±0.3a | 1.6±0.1a | _ | 1.4±0.2a | 2.2±0.3b | _ |
| 1506.6 | Decanal | 1498.0 | 4.0±0.7a | 7.3±0.3a | 13.9±0.4a | 5.1±0.1a | 3.8±0.4a | 4.0±0.2a | 6.0±0.5a | 6.0±0.7a | 5.3±0.1a | 5.3±0.3a |
| 1544.0 | Benzaldehyde | 1520.0 | 6.1±0.3a | 7.7±0.4a | 14.4±0.8b | 7.3±1.0a | 8.0±0.9a | 7.4±0.2a | 7.1±0.1a | 8.4±0.2a | 4.8±0.2a | 6.9±0.3a |
| 1548.8 | (E)-2-Nonenal | 1534.0 | 2.3±0.1a | 2.6±0.4a | 2.5±0.4a | 2.2±0.1a | 2.2±0.1a | 1.9±0.3ab | 1.5±0.3b | 2.2±0.48a | 2.6±0.1a | 1.4±0.2b |
| 1613.4 | Undecanal | 1604.0 | 0.6±0.1a | 0.7±0.1a | 1.4±0.1b | _ | 0.4±0.1a | 0.4±0.1a | _ | 0.6±0.1a | 0.6±0.1a | _ |
| 1396.2 | 2-Butenal | 1047.0 | _ | _ | _ | _ | _ | _ | _ | _ | _ | 54.9±4.5 |
|  | **Phenols** |  |  |  |  |  |  |  |  |  |  |  |
| 2106.3 | 2,4-Di-tert-butylphenol | 2318.0 | 8.1±0.6a | 2.7±0.1ab | 0.9±0.1a | 6.9±0.1b | 3.6±0.6a | 2.2±0.1a | 12.3±0.1b | 5.0±0.1b | 2.3±0.1a | 2.9±0.1ab |

Compounds identified via GC/MS analysis based on comparison with the R.I. (Retention Indices) and the mass spectra of standard compounds (similarity ≥ 85% were listed). Mean values with different lower-case letters in the same row correspond to significant differences at *P* < 0.05. “–”: Not detected.

Table S2 Sensory evaluation score table of winter jujube during storage at 20 ^o^C and 2 ^o^C.

| Sensory evaluation | Packaging |  | 20 ^o^C | | | | | | 2 ^o^C | | | | | | |
| --- | --- | --- | --- | --- | --- | --- | --- | --- | --- | --- | --- | --- | --- | --- | --- |
|  |  |  | Storage time | | | | | | | | | | | | |
|  |  |  | 0 | 1 | 2 | 3 | 4 | 5 | | 0 | 7 | 14 | 21 | 28 | 35 |
| Crunchiness | MP-MAP |  | 8±0.46a | 8±0.43a | 8±0.47a | 8±0.46a | 7±0.56a | 7±0.53a | | 8±0.68a | 8±0.71a | 7±0.68a | 7±0.54a | 5±0.34a | 4±0.68a |
|  | PMP-MAP |  | 8±0.35a | 8±0.41a | 8±0.44a | 8±0.47a | 8±0.51b | 8±0.51b | | 8±0.67a | 8±0.25a | 8±0.64b | 7±0.65a | 6±0.54b | 5±0.57b |
|  | NP-MAP |  | 8±0.46a | 7±0.38b | 6±0.51b | 5±0.21b | 5±0.47c | 4±0.61c | | 8±0.84a | 7±0.48b | 7±0.55a | 3±0.58b | 3±0.36c | 2±0.64c |
| Juiciness | MP-MAP |  | 8±0.45a | 8±0.47a | 8±0.51a | 7±0.52a | 7±0.51a | 7±0.63a | | 8±0.85a | 7±0.71a | 7±0.67a | 6±0.81a | 6±0.61a | 5±0.51a |
|  | PMP-MAP |  | 8±0.48a | 8±0.44a | 8±0.52a | 8±0.48b | 8±0.48b | 7±0.54a | | 8±0.71a | 8±0.38b | 8±0.74b | 7±0.25b | 6±0.52b | 6±0.34b |
|  | NP-MAP |  | 8±0.65a | 7±0.42b | 7±0.43b | 6±0.48c | 6±0.47c | 5±0.21b | | 8±0.87a | 7±0.68a | 5±0.67c | 5±0.64a | 4±0.47c | 3±0.38c |
| Sweetness | MP-MAP |  | 8±0.66a | 8±0.64a | 8±0.78a | 8±0.54a | 8±0.67a | 7±0.68a | | 8±0.64a | 8±0.71a | 7±0.54a | 6±0.71a | 6±0.38a | 5±0.61a |
|  | PMP-MAP |  | 8±0.56a | 8±0.62a | 8±0.71a | 8±0.64a | 8±0.57a | 7±0.81a | | 8±0.57a | 8±0.54a | 8±0.24b | 7±0.35b | 6±0.64a | 5±0.57b |
|  | NP-MAP |  | 8±0.48a | 7±0.36b | 6±0.56b | 6±0.67b | 6±0.88b | 5±0.65b | | 8±0.64a | 7±0.68b | 4±0.68c | 3±0.72c | 2±0.54b | 1±0.36c |
| Flavor | MP-MAP |  | 8±0.55a | 8±0.41a | 8±0.47a | 8±0.88a | 7±0.87a | 7±0.34a | | 8±0.57a | 8±0.71a | 8±0.54a | 7±0.87a | 7±0.71a | 6±0.54a |
|  | PMP-MAP |  | 8±0.62a | 8±0.52a | 8±0.48a | 8±0.46a | 8±0.67b | 7±0.75a | | 8±0.81a | 8±0.67a | 7±0.67b | 5±0.65b | 5±0.65b | 4±0.64b |
|  | NP-MAP |  | 8±0.63a | 7±0.36b | 6±0.34b | 4±0.41b | 3±0.54c | 1±0.57b | | 8±0.38a | 3±0.72b | 2±0.58c | 1±0.66c | 1±0.54c | 1±0.24c |
| Visual appearance | MP-MAP |  | 8±0.67a | 8±0.48a | 8±0.26a | 7±0.46a | 6±0.24a | 6±0.65a | | 8±0.72a | 8±0.54a | 8±0.57a | 6±0.47a | 5±0.61a | 4±0.36a |
|  | PMP-MAP |  | 8±0.54a | 8±0.48a | 8±0.61a | 7±0.54a | 7±0.36b | 7±0.75b | | 8±0.75a | 8±0.67a | 8±0.67a | 7±0.65b | 6±0.24b | 6±0.45b |
|  | NP-MAP |  | 8±0.36a | 8±0.55a | 7±0.64b | 6±0.64b | 6±0.61a | 5±0.67c | | 8±0.74a | 8±0.87a | 6±0.57b | 4±0.67c | 3±0.62c | 2±0.54c |

Means ± SE followed by different letters in the same column for the same evaluated parameter are significantly different (*P <* 0.05) according to the LSD test.

Table S3 Scoring criteria for the attributes considered in this work.

| Descriptors | Scores and description | | | |
| --- | --- | --- | --- | --- |
|  | 1-2 | 3-4 | 5-6 | 7-8 |
| Crunchiness | Loose texture, very poor taste, mouth food more slag | Loose texture, taste average, less dregs in mouth | More tender, the meat is crisp and Good taste, no crumb in mouth | Crispy meat, excellent taste excellent, no crumbs in the mouth |
| Juiciness | Very little juice | Less juice | The juice is medium | Tender and juicy |
| Sweetness | The pulp has almost no sweetness and is very sour | Flesh is lighter in sweetness and heavier in acidity | Flesh is lighter in sweetness and heavier in acidity | Flesh is very sweet, no other taste |
| Flavor | The date flavor is very light, accompanied by a strong wine flavor | Date flavor is relatively light, with a light faint smell of wine | Lighter flavor of winter jujube, no wine flavor | Strong date flavor, no wine flavor |
| Visual appearance | Rougher skin, small holes on the surface, yellow coloring, browning-like phenomenon | Rougher skin, poorer fruit shape, darker coloring | Smooth skin, slightly poor appearance, coloring average | Smooth skin, slightly poor appearance, coloring average |

Table S4 Two-way NAOVA of storage time and packaging on weight loss, TSS, TA, firmness, decay incidence of winter jujube at 20 ℃ and 2 ℃.

| 20 ℃ |  | Physicochemical parameters | | | | |  |
| --- | --- | --- | --- | --- | --- | --- | --- |
|  |  | Weight loss (%) | TSS (%) | TA (%) | Firmness (N) | Decay incidence (%) |  |
| Two-way ANOVA |  |  |  |  |  |  | |
| Storage times (S) |  | *P* < 0.001 | *P* < 0.001 | *P* = 0.691 | *P* < 0.001 | *P* < 0.001 |  |
| Packaging (P) |  | *P* = 0.001 | *P* < 0.001 | *P* < 0.001 | *P* < 0.001 | *P* < 0.001 |  |
| S × M |  | *P* < 0.001 | *P* < 0.001 | *P* < 0.006 | *P* < 0.001 | *P* < 0.001 |  |

| 2 ℃ |  | Physicochemical parameters | | | | |
| --- | --- | --- | --- | --- | --- | --- |
|  |  | Weight loss (%) | TSS (%) | TA (%) | Firmness (N) | Decay incidence (%) |
| Two-way ANOVA |  |  |  |  |  |  |
| Storage times (S) |  | *P* < 0.001 | *P* < 0.001 | *P* < 0.001 | *P* < 0.001 | *P* < 0.001 |
| Packaging (P) |  | *P* < 0.001 | *P* < 0.039 | *P* = 0.005 | *P* < 0.003 | *P* < 0.001 |
| S × M |  | *P* < 0.001 | *P* = 0.161 | *P* < 0.001 | *P* < 0.902 | *P* < 0.001 |

The influence factors cultivar, maturity and their interactions were analyzed using two-way ANOVA.

Table S5 Effect of winter jujube on TPC, TFC, ABTS, DPPH, CUPAC and FRAP of winter jujube along with storage time.

Correlation analysis of indicators of antioxidant ability are recorded as follow (n = 3).

| Index | TPC | TFC | ABTS | DPPH | CUPAC | FRAP |
| --- | --- | --- | --- | --- | --- | --- |
| TPC | 1 |  |  |  |  |  |
| TFC | .734** | 1 |  |  |  |  |
| ABTS | -0.228 | -0.103 | 1 |  |  |  |
| DPPH | -0.077 | -0.266 | 0.094 | 1 |  |  |
| CUPAC | .816** | .849** | -0.037 | -0.186 | 1 |  |
| FRAP | .728** | .952** | -0.172 | -0.152 | .868** | 1 |

Note:* and ** mean significant correlation at *P* < 0.05 and extremely significant correlation at *P*< 0.01. respectively.
